# Crosstalk Between Microbiota, Microbial Metabolites, and Interferons in the Inflammatory Bowel Disease Gut

**DOI:** 10.1093/jcag/gwad044

**Published:** 2023-12-25

**Authors:** Vi To Diep Vu, Ramsha Mahmood, Heather K Armstrong, Deanna M Santer

**Affiliations:** Department of Immunology, University of Manitoba, 429 Apotex Centre, Winnipeg, MB, R3E 0T5Canada; Children’s Hospital Research Institute of Manitoba, University of Manitoba, 715 McDermot Avenue, Winnipeg, MB, R3E 3P4Canada; Manitoba IBD Clinical and Research Centre, University of Manitoba, Winnipeg, 820 Sherbrook St, MB, R3A 1R9Canada; Department of Internal Medicine, Manitoba Center for Proteomics and System Biology, University of Manitoba, 715 McDermot Ave, Winnipeg, MB, R3E 3P4Canada; Department of Immunology, University of Manitoba, 429 Apotex Centre, Winnipeg, MB, R3E 0T5Canada; Children’s Hospital Research Institute of Manitoba, University of Manitoba, 715 McDermot Avenue, Winnipeg, MB, R3E 3P4Canada; Manitoba IBD Clinical and Research Centre, University of Manitoba, Winnipeg, 820 Sherbrook St, MB, R3A 1R9Canada; Department of Internal Medicine, Manitoba Center for Proteomics and System Biology, University of Manitoba, 715 McDermot Ave, Winnipeg, MB, R3E 3P4Canada; Department of Immunology, University of Manitoba, 429 Apotex Centre, Winnipeg, MB, R3E 0T5Canada; Children’s Hospital Research Institute of Manitoba, University of Manitoba, 715 McDermot Avenue, Winnipeg, MB, R3E 3P4Canada; Manitoba IBD Clinical and Research Centre, University of Manitoba, Winnipeg, 820 Sherbrook St, MB, R3A 1R9Canada; Department of Internal Medicine, Manitoba Center for Proteomics and System Biology, University of Manitoba, 715 McDermot Ave, Winnipeg, MB, R3E 3P4Canada; Department of Medical Microbiology and Infectious Diseases, University of Manitoba, 745 Bannatyne Ave, Winnipeg, MB, R3E 0J9Canada; Department of Food and Human Nutritional Sciences, University of Manitoba, 35 Chancellor’s Circle, Winnipeg, MB, R3T 2N2Canada; Department of Immunology, University of Manitoba, 429 Apotex Centre, Winnipeg, MB, R3E 0T5Canada; Children’s Hospital Research Institute of Manitoba, University of Manitoba, 715 McDermot Avenue, Winnipeg, MB, R3E 3P4Canada; Manitoba IBD Clinical and Research Centre, University of Manitoba, Winnipeg, 820 Sherbrook St, MB, R3A 1R9Canada

## Abstract

With the prevalence of inflammatory bowel diseases (IBD) continuing to rise in Canada and globally, developing improved therapeutics that successfully treat greater percentages of patients with reduced complications is paramount. A better understanding of pertinent immune pathways in IBD will improve our ability to both successfully dampen inflammation and promote gut healing, beyond just inhibiting specific immune proteins; success of combination therapies supports this approach. Interferons (IFNs) are key cytokines that protect mucosal barrier surfaces, and their roles in regulating gut homeostasis and inflammation differ between the three IFN families (type I, II, and III). Interestingly, the gut microbiota and microbial metabolites impact IFN-signaling, yet how this system is impacted in IBD remains unclear. In this review, we discuss the current knowledge of how gut microbiota directly or indirectly impact IFN levels/responses, and what is known about IFNs differentially regulating gut homeostasis and inflammation in animal models or patients with IBD.

## Introduction

Inflammatory bowel diseases (IBD), Crohn disease (CD), and ulcerative colitis (UC) affect ~320,000 Canadians, negatively impacting quality of life and incurring significant financial impacts on patients, their families, and the healthcare system (>$5 billion total/year in Canada).^1^ Existing therapies target specific immune pathways, yet not every patient responds, many have adverse side effects, and some patients develop intolerance over time.^2^ Improving our understanding of the key immune pathways involved in IBD is essential to identify novel therapeutic targets and to advance the treatment paradigm.

## Interferon biology

**Figure 1.**
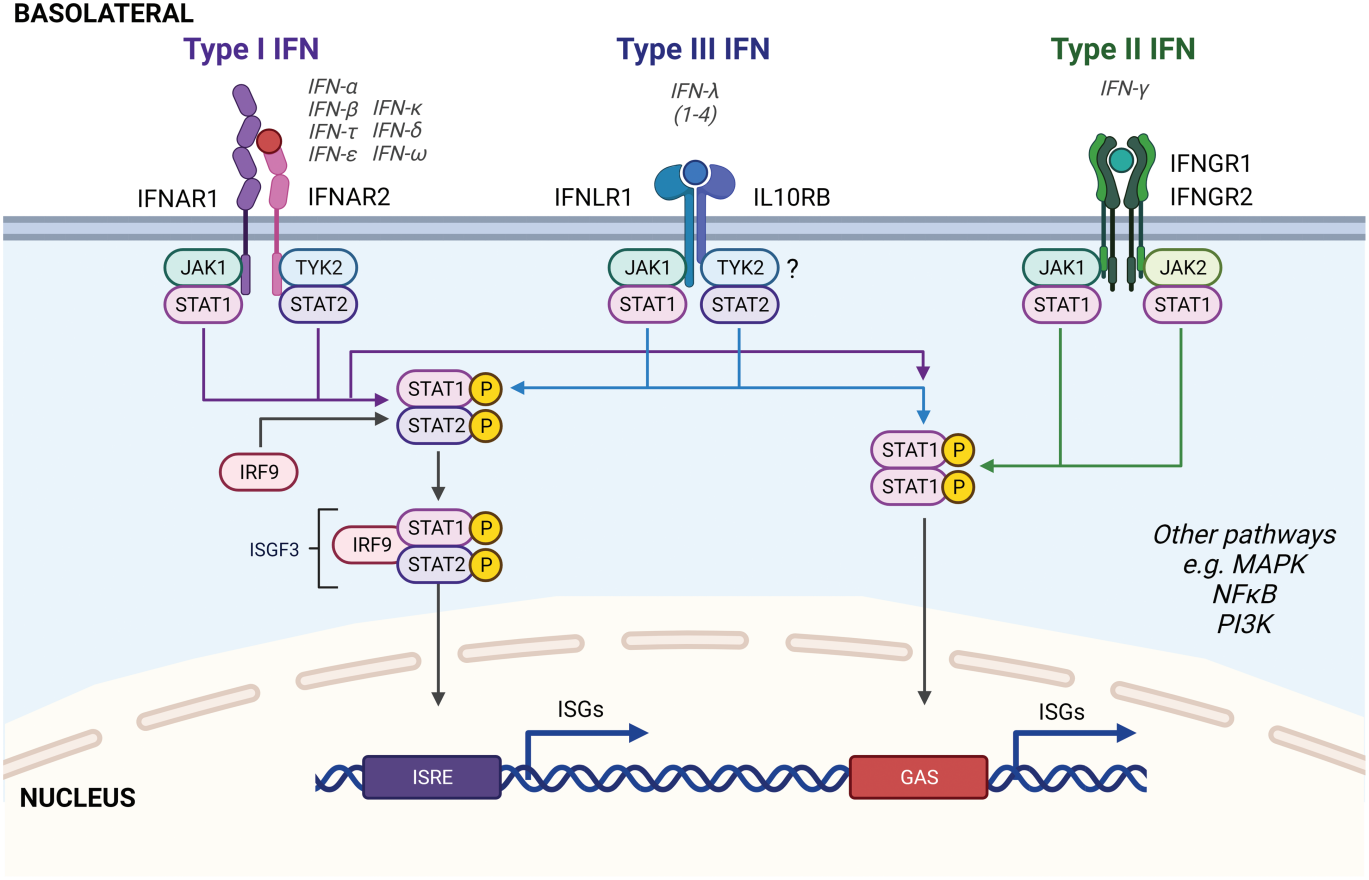
Overview of the three IFN receptor signaling pathways. In the human intestine, the majority of all IFN receptors are located basolaterally. Signaling downstream of each heterodimeric receptor involves the activation of janus kinases (JAKs; JAK1, JAK2, TYK2) and phosphorylation of signal transducer and activator of transcription-1 or -2 (STAT1/2). The ? by TYK2 in the type III IFN pathway reflects recent studies demonstrating TYK2 independent signaling is also possible in certain cell types.^12^ In type I and III IFN signaling, activated STAT1/2 binds to interferon regulatory factor 9 (IRF9), forming the IFN-stimulated gene factor 3 (ISGF3) transcriptional complex that translocates to the nucleus to promote expression of hundreds of IFN-stimulated genes (ISGs) through binding IFN-stimulated response elements (ISREs). The alternative JAK/STAT pathway shown involves the formation of phosphorylated STAT1 homodimers that translocate to the nucleus to bind gamma interferon-activated sites (GAS) to induce the expression of a distinct set of ISGs, some of which are pro-inflammatory in IBD. This is a simplified figure as there are also other STATs and other signaling cascades activated downstream of each IFN family receptor including for example mitogen‑activated protein kinase (MAPK), phosphatidylinositol 3-kinase (PI3K)/Akt, and nuclear factor-κB (NFκB). TYK2, tyrosine kinase 2; P, phosphate. Created with BioRender.com.

Interferons (IFNs) are critical innate immune cytokines that were initially discovered for their potent antiviral activity as early as 1957.^3^ They are classified into three families: type I (IFN-α, IFN-β, etc.), type II (IFN-γ), and type III (IFN-λ1-4), with type III IFNs the most recently identified in 2003 (IFN-λ1-3) and 2013 (IFN-λ4).^3–7^ Each IFN family interacts with their own unique heterodimeric receptor that differ in their cell distribution. In this review, we group type I and III together in each discussion due to their similar induction mechanisms which are distinct from the type II IFN IFN-γ. Type I IFNs signal via IFNAR1/IFNAR2 on every nucleated cell, type II IFNs signal via IFNGR1 which is ubiquitously expressed on all cell types, and IFNGR2 which is found especially on immune cells, but also on some mucosal barrier cells.^8^ Type III IFNs signal via IFNLR1/IL10RB where IL10RB is ubiquitously expressed, and IFNLR1 is found only on epithelial cells and specific immune cell types at varying levels; dendritic cells, macrophages, B cells, and neutrophils respond to IFN-λs, although there are differences in IFN-λ immune responsive cell types between mice and humans.^4,8–11^ All three IFN families induce gene expression through the canonical Janus kinase–signal transducer and activator of transcription (JAK–STAT) signaling pathway (**fig. 1**; reviewed in ^13–15^). In brief, receptor associated kinases (JAK1/2 (or TYK2 Type I/III)) are activated and promote a signaling cascade that leads to the phosphorylation of STAT1 and/or STAT2. Phosphorylated STAT1 homodimers bind to conserved IFN-γ activation site (GAS) DNA elements or the ISGF3 complex comprised of phosphorylated STAT1, STAT2 and interferon regulatory factor 9 (IRF9) binds to IFN-stimulated response elements (ISREs) to activate transcription of hundreds of ISGs (reviewed in ^16^). Many up- and down-regulated genes overlap among the IFN families, especially when studying antiviral immunity, but unlike type I and II IFNs, type III IFNs have been shown to act without inflammatory side effects.^17–19^ This could be related to type III IFNs being more structurally similar to the important anti-inflammatory and gut barrier promoting IL-10 family of cytokines (e.g., IL-10, IL-22, IL-24, and IL-26).^20,21^ Other pathways are also activated downstream of each IFN receptor that work in parallel or cooperate with the JAK/STAT pathways such as mitogen‑activated protein kinase (MAPK), phosphatidylinositol 3-kinase (PI3K)/Akt, and nuclear factor-κB (NFκB).^8,22–25^

**Figure 2.**
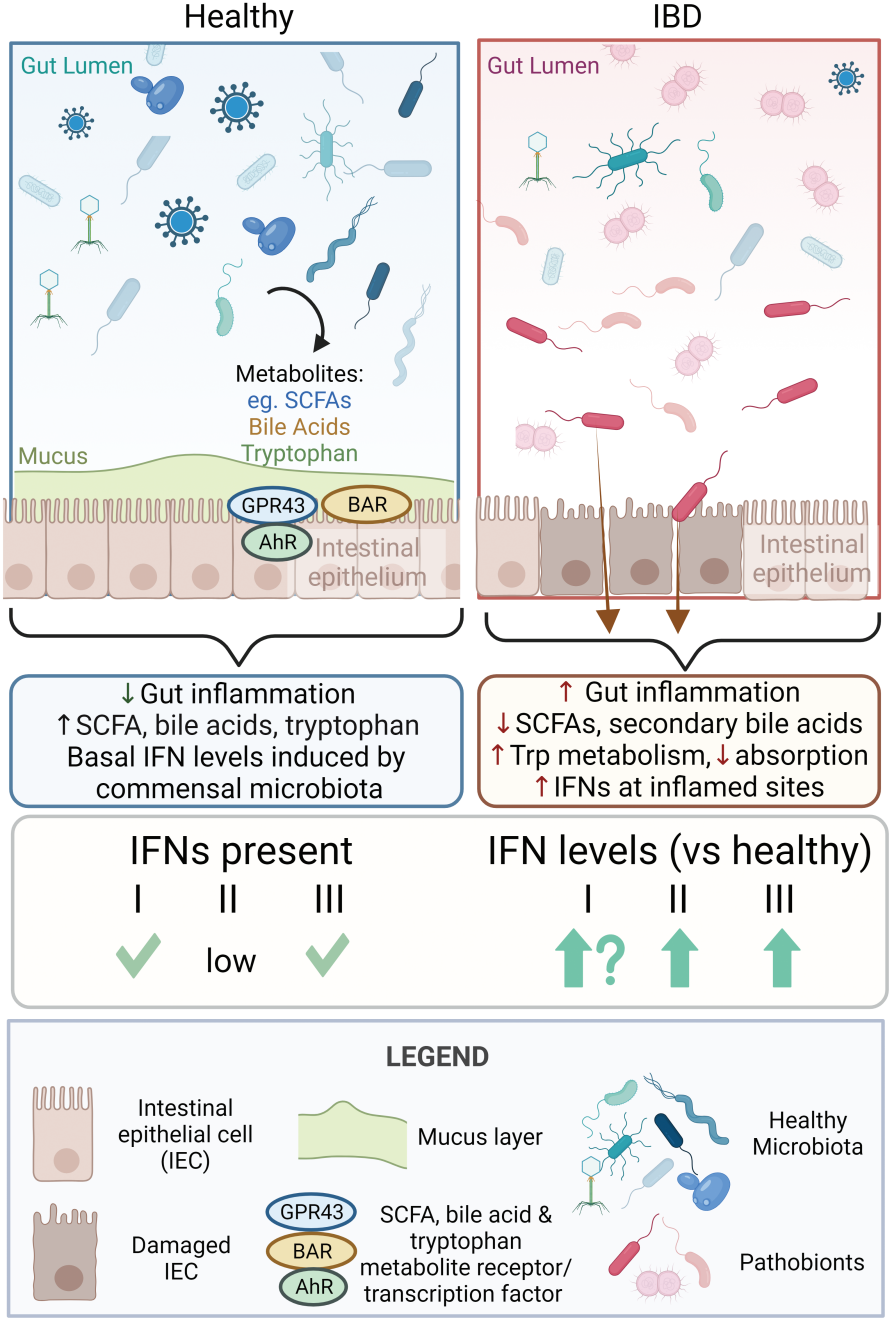
Comparison of gut microbiota and downstream metabolites in the regulation of interferons in healthy versus inflammatory bowel disease tissue. Summary of what is known and discussed in this review related to steady state induction of interferons (IFNs) by microbiota and metabolites, and how this is potentially altered in inflammatory bowel disease (IBD) patients. Trp, tryptophan; AhR, aryl hydrocarbon receptor; SCFA, short-chain fatty acids; BAR, bile acid receptor. Created with BioRender.com.

IFNs are well known for their roles in inducing antimicrobial (bacterial, fungal, viral) immune responses, but growing evidence demonstrates each family also distinctly affects gut homeostasis and inflammatory pathways.^26–28^ Recent reviews have focused attention on the latest findings for the roles of IFNs inhibiting pathogens at barrier sites^29–31^ and regulating IBD pathogenesis.^32,33^ This review will outline how gut microbiota and downstream metabolites directly and indirectly affect each IFN family and how this relates to gut health as related to IBD (**fig. 2**).

## Direct microbiota interactions modulating IFNs

The first step of type I and III IFN induction involves interactions of pathogen-associated molecular patterns or microbe-associated molecular patterns and pattern recognition receptors (PRRs).^34,35^ Specifically in the gut, type I and III IFN expression is driven through direct interactions of commensal microbiota or enteric pathogens (virus and bacteria) with PRRs such as toll-like receptors (TLRs), nucleotide-binding oligomerization domain (NOD)-like receptors, C-type lectin receptors, and retinoic acid-inducing gene I (RIG-I)-like receptors.^36–39^ Cell-type-specific responses occur due to variable PRR and transcription factor levels that directly regulate IFN expression (e.g., IFN regulatory factors (IRFs)).^40^ The cell types responsible for the type I and III IFN production in the gut include both innate immune cells (e.g., dendritic cells, macrophages) and epithelial cells.^40,41^ Microbes can also induce IFN induction by causing inflammatory cell death (e.g., pyroptosis, necrosis) or damage to gut cells leading to the release of danger-associated molecular patterns that are also recognized by PRRs (reviewed in^42,43^).

The induction and signaling of IFNs in the gut in the context of pathogens have been reviewed recently,^26,31,44^ although less is known regarding homeostatic IFN signaling. Germ-free mice or treatment of mice with antibiotics significantly decreased intestinal IFN induction, demonstrating the importance of commensal microbiota for baseline IFN levels contributing to gut homeostasis and also protection from enteric viral infections.^45,46^ This is not always the case though, as some enteric viruses exploit commensal bacteria to enhance infection.^47^ While gut commensal bacteria induce IFNs, the gut virome is also a major player in driving IFN expression and even regulating gut inflammation.^48–50^ Evidence is only starting to emerge for IFN induction by fungi or helminths present in the gut.^51,52^ Spatially, type III IFNs were found to be more potent for signaling in intestinal epithelial cells, whereas type I IFNs especially stimulate lamina propria immune cells.^41,46^

Unlike type I and III IFNs, where initial microbes are sensed especially by epithelial cells and dendritic cells, the type II IFN, IFN-γ, is produced mainly by immune cells in the gut such as NK cells, T cells, and type I innate lymphoid cells via indirect interactions with microbiota.^27,53,54^ IFN-γ is known as a pro-inflammatory cytokine that has multiple functions in the gut for directly promoting pathogen-specific immunity and regulating homeostatic processes. This occurs through actions on immune cells (e.g., macrophages, natural killer cells, and T cells), epithelial cells and stem cells.^28,55,56^ For many years, IFN-γ has been known to directly impact pathological mechanisms in IBD and in mouse models through promoting gut barrier dysfunction and intestinal inflammation.^27,28,57,58^ IFN-γ is one of the core cytokines secreted by immune cells in the lamina propria during active IBD, although a more pronounced IFN-γ phenotype is likely to be detected in active CD, compared to UC.^59–62^

## Microbiota indirectly impact IFNs via metabolites

There has been an increasing focus on how gut microbiota regulate gut homeostasis, barrier integrity and intestinal immune responses through metabolites. The multitude of effects of microbiota metabolites on the immune system and gut inflammation are discussed elsewhere.^37,63,64^ Here, we will focus on regulation of IFNs by a few example metabolites directly produced by microbiota, altered host cell metabolites, and small molecules derived from microbiota metabolism of dietary substrates. Since the gut microbiota community and metabolome are significantly altered in IBD,^65,66^ changes in intermediate or end products of microbial metabolism could impact gut IFN levels. There are three key classes of microbiota-associated metabolites that are commonly acknowledged for their role in gut homeostasis and inflammation in IBD, including short-chain fatty acids (SCFAs), bile acids, and tryptophan metabolites.^66^

SCFAs are produced when bacteria metabolize dietary carbohydrates.^67^ Recently, SCFAs have been shown to directly impact IFN levels in the gut, or in distal mucosal sites such as the lung.^68^ Butyrate and acetate improved viral clearance in multiple animal models by inducing type I and type III IFNs through interactions with the SCFA receptor GPR43.^69–73^ The effects of butyrate on IFN-γ responses are context dependent; butyrate has been shown to directly inhibit IFN-γ (and type I IFN) JAK/STAT signaling, but yet elevated butyrate levels also promote inflammatory pathways during acute colitis in an animal model via IFN-γ producing T cells.^74–76^ Therefore, SCFAs may be beneficial or detrimental within the gut, promoting inflammation when needed (infection), but also when not (autoimmune disease), dependent on the levels of SCFAs present and their interactions with different IFN pathways. Decreases in SCFA levels, especially butyrate and propionate, are common in IBD, particularly during disease flare, and this is associated with lower SCFA-producing bacteria in the gut.^77,78^ Continued research is needed to uncover how the balance between SCFA levels differentially impacts each IFN family, and how this process could be manipulated in disease settings and infection.

Primary bile acids are modified by gut microbiota into secondary bile acids that have been reported to upregulate type I and type III induction in intestinal tissues, although this was studied in the context of viral infections.^79,80^ Microbiota-derived secondary bile acids can interact with multiple bile acid receptors to modulate various immune pathways in the gut,^81^ but the mechanism of bile acid signaling upregulating IFN induction is not fully understood. An opposite, antagonizing effect on type I/II IFN signaling was reported for bile acids on liver cells^82,83^ and bile acid signaling in macrophages decreased IFN-γ production.^84,85^ Bile acid absorption is decreased and altered bile acid metabolism has been reported in active IBD. In particular, primary bile acids such as chenodeoxycholic acid are higher in IBD patients, while secondary bile acids such as lithocholic acid and deoxycholic acid are lower.^86^ Lithocholic acid is known to inhibit Th1 activation and decrease IFN-γ production by CD4+ T cells.^87^ The role of secondary bile acids and type I and III IFNs in IBD should be studied in more detail.

Tryptophan is an important amino acid that is metabolized by both host enzymes and the gut microbiota into various metabolites that regulate multiple processes in the gut^88^ IBD patients can have lower systemic tryptophan levels and decreased tryptophan absorption in the gut where lower tryptophan levels could contribute to development of IBD or worsen disease activity.^89^ Tryptophan metabolites signal through the ligand binding transcription factor aryl hydrocarbon receptor (AhR) to downregulate gut inflammation in mouse models, which can include downregulating IFN-γ signaling^90,91^ As well, IFN-γ can promote tryptophan metabolism by upregulating indoleamine-2,3-dioxygenase levels.^92^ AhR levels can also be downregulated in IBD patient intestinal tissue.^90^ Less is known about tryptophan metabolite crosstalk with type I and III IFNs in the gut.

Two other metabolites have been implicated in regulating IFN levels in recent studies, but were studied for their protection of mice for viral infection.^93,94^*Clostridium butyricum* ω-3 fatty acid 18-HEPE production led to upregulated IFN-λ levels in the lung.^93^ Similarly, the metabolite desaminotyrosine promoted type I IFN production to protect mice from lethal infection across the gut-lung axis.^94^ Other alterations in the IBD serum, stool, and mucosal metabolomes are common,^95^ but more studies are needed to determine how the various metabolites affect IFN levels and signaling in the human gut and how this relates to IBD.

## The impact of IFNs on gut health and IBD

Type I and III IFNs have been predominantly studied in the gut for their critical roles in antiviral immune responses, including against norovirus and rotavirus.^96–100^ There is now increasing evidence both IFN families also regulate gut homeostasis and inflammation in IBD and mouse colitis models, although not necessarily through the same mechanisms due to differential receptor distribution and signaling.^26,32,101^ Type I IFNs can signal through the ubiquitous IFNAR1/IFNAR2 heterodimeric receptor present on any immune cell present in the gut, but type III IFNs regulate inflammatory pathways in the gut through neutrophils or signaling in gut epithelial cells directly to regulate gut barrier function and healing.^102,103^ SNPs that directly impact IFN induction and signaling are linked to IBD, although these can overlap or differ between CD and UC (e.g., *JAK2, TYK2, STAT1, STAT3, IFNGR2, IFIH1*).^104^ Recently, differential type I and type II IFN signatures based on the role of gut microbial composition predicted anti-TNF treatment outcomes for IBD patients.^105^ However, how IFNs and host microbiome interact with each other in terms of IBD pathogenesis and overall gut health still requires additional research.

Type II IFN levels and downstream signaling have been associated with especially CD pathogenesis.^57,60,62,106^ IFN-γ treatment has been shown to negatively impact mucosal epithelial barrier integrity by inducing tight junction disruption.^107^ Studies have used IFN-γ, TNF-α, and IL-1β to mimic IBD intestinal inflammation to study various aspects of epithelial cell function.^108,109^ Furthermore, increased colonic vascular permeability/leakage, and inflammatory cell infiltration upon IFN-γ treatment has also been observed in dextran sulfate sodium (DSS)-induced colitis murine models.^58,110,111^ Of note, not all mouse models of intestinal inflammation are IFN-γ driven/dependent, and inflammation is driven especially by Th1/IFN-γ (and Th17) in CD, but not UC.^33,62,112^

Traditionally, type III IFNs are less inflammatory compared to type I IFNs,^18,113^ although both regulate gut inflammation in animal models. Type I IFNs can limit the severity of gut damage in two colitis mouse models.^101,114^*Ifnlr1* knockout mice had worse intestinal inflammation and damage upon DSS challenge compared to wildtype mice, and treatment of DSS-induced colitis with mouse Ifn-λ improved mucosal healing and dampened inflammation.^102,103^ Type I and type III IFN receptor double knockout mice were also more susceptible to experimental colitis with increased severity and a significant reducition in goblet cells.^115^ In contrast to the protective role of IFN-λ suggested above, IFN-λ expression was upregulated in inflamed tissues from UC and CD patients, and IFN-λ could induce cell death in Paneth cells and small intestinal organoids derived from mice.^103,116^ Therefore, more research is needed to understand the roles of IFNs at steady state, versus during periods of inflammation in both mouse and human samples.

## IFNs as a potential therapeutic target

Given their significance in gut homeostasis and involvement in IBD pathogenesis, studies have looked at IFNs as a potential therapeutic target for IBD patients. In a small study, 12 patients with active CD were given the type I IFN, IFN-α, for 24 weeks.^117^ There was no beneficial effect on inflammatory markers or endoscopic activity and patients experienced side effects from the treatment. Years later, two multicentre placebo controlled trials tested IFN-α or IFN-β as a potential therapy for UC,^118,119^ which presents with a more typical Th2 phenotype that type I IFNs were shown to regulate in other settings.^62,112,120^ Unfortunately, no significant clinical benefit was observed between the placebo group and the group receiving either IFN treatment, suggesting the doses tested were not an effective option for UC patients. Fontolizumab, a humanized anti-IFN-γ antibody treatment, has also been administered to patients with moderate to severe CD, in phase I and II placebo controlled, double-blind clinical trials.^121,122^ In both trials, there was no statistical significance in treatment response at the primary end point, though a significant increase in clinical response at a later timepoint was found in the larger phase II trial. IFN-λs have not been targeted in IBD directly yet in any clinical trial. JAK inhibitors (JAKi) were recently developed to block the signaling of various inflammatory cytokines implicated in IBD pathogenesis (reviewed in ^123^). Since all three IFN families signal through the JAK/STAT pathway (**fig. 1**), the JAKi tofacitinib administered to moderate to severe UC patients would likely inhibit both the detrimental (e.g., IFN-γ) or potential beneficial (e.g., IFN-λ) IFN activity known to occur in the gut.^13^ Newer JAK1-specific inhibitors have promising results as a future treatment for CD and TYK2 inhibitors are also now being tested which would more specifically inhibit IL-12, IL-23, type I, and type III IFN signaling.^123,124^ Finding the perfect balance to block the inflammatory immune response can be difficult in IBD, but more selective inhibitors are being developed and using these alone or in combination with, for example, microbiota-targeted therapies, could benefit a greater number of IBD patients.

## Conclusions

Aberrant mucosal immune responses, external exposures, and alterations of the gut microbiome have been proposed as contributing risk factors of IBD, but *specific* immune pathways need to be elucidated to design targeted therapeutic approaches.^125,126^ The role of IFN signaling in modulating the host mucosal immune response and how gut microbiota alter this is still a growing area of research.

Given the significant impact that steady state and aberrant IFN signaling can have on the mucosal immune response in IBD, more research is needed to fully understand the interactions between gut microbes and IFNs, but also between IFN families themselves. Each IFN family is distinct when it comes to their roles in altering gut inflammation, epithelial barrier integrity, and mucosal healing and this will likely vary between UC and CD. Studying gut microbiota and metabolites could also provide additional therapeutic targets, where some directly or indirectly affect IFN and key inflammatory pathways implicated in IBD pathogenesis. Re-establishing the balance of the gut microbiota community in IBD patients is a core focus of therapeutic discovery in this field, which will likely affect IFNs and other innate cytokines. New therapeutic angles are needed for IBD patients when current immune targeting therapies do not always work or cause significant side effects in many patients. Thinking beyond blocking/inhibiting the gut immune system in IBD is necessary, taking into account the crosstalk between an altered microbiome and metabolome in IBD and key immune regulatory cytokines such as IFNs.

## Data Availability

There are no data associated with this manuscript.
